# Revisiting blood transfusion and predictors of outcome in cardiac surgery patients: a concise perspective

**DOI:** 10.12688/f1000research.10085.1

**Published:** 2017-02-20

**Authors:** Carlos E Arias-Morales, Nicoleta Stoicea, Alicia A Gonzalez-Zacarias, Diana Slawski, Sujatha P. Bhandary, Theodosios Saranteas, Eva Kaminiotis, Thomas J Papadimos

**Affiliations:** 1 Department of Anesthesiology, The Ohio State University Wexner Medical Center, Columbus, OH, USA; 2 College of Medicine and Life Sciences, The University of Toledo, Toledo, OH, USA; 3 Second Department of Anesthesiology, School of Medicine, University of Athens, Athens, Greece

**Keywords:** blood transfusion, cardiopulmonary bypass, cardiac surgery, length of stay, mortality

## Abstract

In the United States, cardiac surgery-related blood transfusion rates reached new highs in 2010, with 34% of patients receiving blood products. Patients undergoing both complex (coronary artery bypass grafting [CABG] plus valve repair or replacement) and non-complex (isolated CABG) cardiac surgeries are likely to have comorbidities such as anemia. Furthermore, the majority of patients undergoing isolated CABG have a history of myocardial infarction. These characteristics may increase the risk of complications and blood transfusion requirement. It becomes difficult to demonstrate the association between transfusions and mortality because of the fact that most patients undergoing cardiac surgery are also critically ill. Transfusion rates remain high despite the advances in perioperative blood conservation, such as the intraoperative use of cell saver in cardiac surgery. Some recent prospective studies have suggested that the use of blood products, even in low-risk patients, may adversely affect clinical outcomes. In light of this information, we reviewed the literature to assess the clinical outcomes in terms of 30-day and 1-year morbidity and mortality in transfused patients who underwent uncomplicated CABG surgery.

## Introduction

According to the National Blood Collection & Utilization Survey (NBCUS), blood transfusion rates, with the exception of platelet transfusions, decreased between 2008 and 2011 ^1^. Specifically, there was an 8.2% decrease in red blood cell (RBC) transfusions as well as a 13.4% decrease in plasma transfusions. In the United States, however, cardiac surgery-related blood transfusion reached new highs in 2010, with 34% of patients receiving blood products ^2^. Cardiac surgeries generate the greatest need for blood transfusion, followed by orthopedic surgeries ^3, 4^. Transfusion is often necessary in cardiac procedures to correct coagulopathy, blood loss, and hemodilution from pump priming ^5^. Furthermore, patients undergoing cardiac surgeries are likely to have comorbidities such as anemia and history of myocardial infarction (MI), thereby increasing the risk of complications and blood transfusion requirement ^2, 3, 6^. It is important to note that the majority of cardiac surgery patients who are transfused and show a high morbidity and mortality receive approximately two units of blood ^3, 7^. These patients are likely being treated for anemia and are otherwise hemodynamically stable ^7– 9^. However, patients in the high-risk subset, based on the European System for Cardiac Operative Risk Evaluation (EuroSCORE) >8, consumed blood products owing to hemorrhagic complications ^3^. In light of this information, we reviewed the literature to assess the 30-day and 1-year morbidity and mortality outcomes in transfused patients who underwent complicated and uncomplicated coronary artery bypass grafting (CABG) surgery defined as the absence of postoperative complications by the Society of Thoracic Surgeons (STS).

## Blood transfusion in critically ill patients

Anemia is a common complication in critically ill patients ^10^. According to the World Health Organization (WHO), anemia is defined as a hemoglobin level of less than 13 g/dL in men and 12 g/dL in women ^11^. There is a complex relationship between RBC transfusions and clinical outcome in critically ill patients. The increased mortality seen in transfused patients might be attributed to a complex illness and might not be related to blood transfusion per se. Statistical measures controlling for mortality risk factors such as preoperative hematocrit, age, or comorbidities allow for presumably unbiased comparison between transfused and non-transfused groups ^12^.

Kulier *et al*. ^13^ describe the problematic paradox faced by clinicians treating anemic patients undergoing CABG surgery. While the authors did not dispute that transfusion may be associated with a variety of complications previously reported in the literature, the authors’ own investigation found a significant correlation between preoperative anemia and renal and central nervous system outcomes as well as in-hospital mortality. Increased cardiac adverse events were attributed to an interaction between preoperative anemia and other comorbidities. Thus, there is a paradox: should anemic patients, especially those with compromised compensatory cardiovascular mechanisms, be preoperatively transfused with RBCs, thus incurring one set of possible complications, or should these patients be transfused only during or after surgery as needed, risking the occurrence of another set of adverse events? Effort should always be made to correct anemia without transfusion prior to cardiac surgery, even in patients able to compensate for their anemia with increased stroke volumes; however, a consensus on when hemoglobin levels indicate preoperative transfusion has yet to be firmly established ^13^.

Generally, 95% of patients admitted to the intensive care unit (ICU) for more than 3 days develop anemia, resulting in multiple blood product transfusions ^14^. It is estimated that 50% of these admitted ICU patients receive RBC transfusions during their stay ^15, 16^. Furthermore, the proportion of patients transfused increases by 30% when the ICU length of stay (LOS) exceeds 7 days. These patients receive a mean of 9.5 units of blood products, and it is estimated that two-thirds of these transfusions are not related to acute blood loss ^10, 16, 17^.

Postoperative anemia is common after CABG surgery and is associated with a compromised cardiovascular outcome. The STS recommended a strict transfusion protocol ^18^ for cardiac surgery patients with hemoglobin levels below 7 g/dL, with RBC transfusions being reasonable and lifesaving if the patient exhibits hemodynamic instability ^19^.

Transfusion of RBCs has been recognized as a risk factor for adverse outcomes in cardiac surgery ^20^ and related to patient characteristics such as left ventricular ejection fraction (LVEF) <35%, age >70, preoperative hemoglobin <11 g/dL, insulin-dependent diabetes, emergency surgeries, female gender, impaired renal function (creatinine >1.6 mg/dL), and re-operation ^21^. Pattakos *et al*. ^22^ studied the outcome of patients undergoing cardiac surgery who refused transfusions owing to religious beliefs. The authors included 322 Jehovah’s Witness patients and 87,453 non-Jehovah’s Witness patients. They concluded that patients who refused transfusion had fewer acute complications and shorter length of stay, better 1-year survival, and similar 20-year survival when compared to transfused patients ^23^.

A retrospective study published by Dejam *et al*. ^12^ demonstrated that RBC transfusion improved outcomes in some patients and worsened the clinical course in patients with particular comorbidities, such as cirrhosis, congestive heart failure, diabetes, sepsis, respiratory disease, solid cancer, and hematologic cancer. However, considering that patients undergoing cardiac surgery are often critically ill, it may be difficult to establish the causation between transfusions and mortality.

## Blood transfusion in uncomplicated cardiac patients

Few studies have recorded the effects of blood transfusions in anemic and non-anemic patients who are otherwise stable. A retrospective study carried out by the Cleveland Clinic found that non-anemic patients (hematocrit >25% during bypass) who received intraoperative blood transfusion for CPB surgery experienced longer ventilator support times, LOS, and decreased long-term survival when compared to non-anemic patients or untreated anemia patients ^23^. Blood transfusion in CABG patients with low- to moderate-risk profiles (EuroSCORE <8), postoperative hemoglobin >10 g/dL, minimal postoperative blood loss, and no preoperative morbidities within 24 hours of surgery posed a greater risk for postoperative cardiac events and infections when compared to those who did not receive any blood transfusion ^8^.

Shaw *et al*. ^24^ retrospectively compared the effects of blood transfusion in a cohort stratified according to hematocrit values. The researchers recovered preoperative hematocrit data from transfused and non-transfused cardiac surgery patients. The authors reported a significantly higher 30-day mortality rate for the transfused group when compared with the non-transfused group. These results aligned with the existing evidence of increased mortality in transfused patients ^8, 24– 26^. Studies of blood transfusion in uncomplicated cardiac surgery allow a more objective evaluation of postoperative outcomes ^8, 9, 23, 24, 27^ and indicate that stable transfused patients do not receive a benefit from this practice in terms of LOS, 30-day morbidity, and 1-year mortality ^24, 27, 28^.

A retrospective study published by Schwann *et al*. examined the association between transfusions and mortality among 6,947 patients who underwent CABG. The overall RBC transfusion rate was 33.9%. Postoperative complications were reported in 35.2% of the patients, specifically in patients who received RBC transfusion when compared with the uncomplicated group. The group reporting complications included older females with previous comorbidities. At 30-day and 5-year follow-up survival, the incidence of death was higher in transfused versus non-transfused patients. RBC transfusion increased the risk of long-term cardiac and non-cardiac mortality after CABG ^29^.

## Blood transfusion in complicated cardiac patients

Blood transfusion is a common practice in complicated patients undergoing CABG surgery. Cardiac patients require higher rates of transfusions than do non-cardiac patients ^30^. Complicated patients generally present with advanced heart disease, co-existing diabetes mellitus, end-stage renal disease, or liver dysfunction, postoperative complications yielding a greater LOS and higher mortality rates ^31^.

Cardiogenic shock (CS) is the primary cause of hospital death after acute MI (AMI). Operative mortality of patients who undergo CABG in the setting of AMI-CS ranges from 20% in isolated CABG to 33% in CABG plus valve surgery, reaching 58% in CABG plus ventricular septal repair ^32^. Similarly, Acharya *et al*. ^33^ evaluated outcomes of 5,496 patients with AMI-CS undergoing non-elective CABG from 2005 to 2013. The authors found an intraoperative mortality of 18.1% for patients who received intraoperative blood products (65.2%) in 2013 when compared to 19.3% in 2005 (p<0.001) ^33^.

Gundling *et al*. conducted an 8-year retrospective study and concluded that the necessity of blood product administration was increased in patients with liver cirrhosis undergoing cardiac surgery; the 30-day mortality rate was higher and long-term survival rate lower when compared to patients without liver cirrhosis ^34^.

Furthermore, recent reports have indicated worse outcomes when transfusion is due to preoperative anemia or blood dyscrasias ^33, 34^. In 2014, Engoren *et al*. evaluated late mortality in 922 patients who underwent isolated CABG during a 3.5-year period. They found that patients with preoperative anemia who received intraoperative transfusion had an increased rate of death compared with those without anemia and transfusion ^35^. Similarly, Paone *et al*. concluded that most of the patients who died postoperatively received blood transfusions and presented a prolonged and complicated postoperative evolution ^8^. Additionally, patients diagnosed with immune thrombocytopenia (ITP) undergoing CABG surgery may require corticosteroid treatment before surgery in order to decrease intraoperative and postoperative bleeding. Jubelirer *et al*. reviewed cases of patients with ITP who underwent CABG. From 51 patients, 21 received platelet transfusion and 27 received RBCs intraoperatively. CABG was successfully performed in these patients without the need of preoperative splenectomy or prolongation of hospital stay ^36^.

Perioperative risk factors might affect the length of ICU stay. Generally, older cardiac surgery patients have longer ICU stays because of their existing comorbidities. Azafarin *et al*. determined in a cross-sectional study that cigarette smoking, opioid addiction, preoperative ejection fraction of ≤40%, and intraoperative blood transfusion are predictors for prolonged ICU stay and a higher rate of complications ^31^.

Paone *et al*. used the predicted risk of mortality measures to confirm the hypothesis that preoperative profile played a more dominant role when considering mortality and transfusion rates in patients undergoing cardiac surgery ^8^. Likewise, other studies have reported weak or no associations between transfusion and mortality ^7, 37, 38^. Koster *et al*. ^39^ in a single-center retrospective study demonstrated no association between the transfusion of one to two units of leucocyte-depleted RBCs and 30-day mortality in CABG patients. Haanschoten *et al*. found that patients with a baseline hemoglobin of ≥11.3 g/dL who underwent isolated CABG surgery without the availability of RBCs in the operating room presented a decrease in complications and LOS ^40^.

Shander also commented on the lack of temporality as a major weakness in this area of research and stated that cause and effect cannot be confirmed based on retrospective studies with unknown blood transfusion time and onset of complications ^9^. Though certain complications may arise rapidly following transfusion, other adverse events may develop in the hours, days, and weeks after blood-product administration, making definitive causation more difficult. While in the former case close temporality provides evidence that transfusion was the impetus for the complication, adverse events which occur days or more after transfusion has concluded may be caused by the transfusion, surgical insult or drug administration, patient comorbidity, or antagonism of several factors. Moreover, transfusion threshold recommendations, despite the evidence-based guidelines, remain an indefinite parameter for blood transfusion; therefore, physicians must carefully deliberate the benefits and potential adverse effects when deciding to transfuse ^41^.

## Conclusion

The relationship between transfusion and clinical outcomes in cardiac patients undergoing complicated and uncomplicated CABG remains poorly understood, suggesting a complex interaction among various patient characteristics, demographics, type of procedure, patients’ preoperative status, and the preferences of surgeons and anesthesiologists and may not be the result of transfusions per se. The increased mortality in transfused patients may or may not be triggered by transfusion itself but rather the outcome of transfusion in a more complex perioperative experience. To better elucidate if and to what degree transfusion plays a role in patient complications following coronary procedures, prospective studies controlling for patient demographics and comorbidities, surgical and anesthesia parameters, and transfusion thresholds are needed. With other previously implicated factors excluded or controlled for, a more definitive consensus on transfusion as a causative factor for specific adverse events, which is lacking in the currently published literature, may be possible regardless of temporality. If transfusion is established as an independent factor for complications, then interactions with other comorbidities and patient and surgical factors should be explored to identify those transfusion patients at highest risk for complication.
